# Large fish eggs may lose their edge as temperature rises

**DOI:** 10.1007/s00442-026-05932-3

**Published:** 2026-07-10

**Authors:** Francesca Leggieri, Oscar Nordahl, Markus Zöttl, Hanna Berggren, Petter Tibblin

**Affiliations:** Centre for Ecology and Evolution in Microbial Model Systems (EEMiS), Department of Biology and Environmental Science, Faculty of Health and Life Sciences, Linnaeus University, Stuvaregatan 4, Kalmar, SE-39231 Sweden

**Keywords:** Climate change, Maternal effects, Oxygen limitation, Propagule size, Thermal performance

## Abstract

It is a nearly universal truth that larger fish eggs have increased chances of survival. However, theory predicts that as temperatures rise, larger eggs may suffer the costs of oxygen limitation and lose their advantage altering the optimal life history trade-off between fecundity and offspring size. Whether these effects may happen under the predicted warming of climate change scenarios is still unknown. Here we show that wild *Esox lucius* females lay smaller eggs later in the season when temperatures are higher and that this was associated with a seasonal decline in female body size. Experimentally, we found that larger eggs had higher survival rates at lower temperatures, but this advantage diminished in rising temperatures and disappeared entirely in temperatures (~ 18 °C) that could arise under climate change. The results demonstrate that the optimal egg size is temperature dependent, and that future warming may favor smaller females with smaller, more numerous, eggs that may profoundly affect population dynamics and the evolutionary optima of life-history traits.

**Supplementary Information:**

The online version contains supplementary material available at 10.1007/s00442-026-05932-3.

## Introduction

Climate change is currently altering the thermal regimes of virtually all habitats on Earth, with significant consequences for the behaviour, distribution, and viability of species, populations, and individuals (Tamarin-Brodsky et al. 2020; IPCC 2022; Jørgensen et al. 2022). A central focus for recent research has been to provide insights into how organisms can cope with and adapt to climate change to both predict and manage its consequences (Huey et al. 2012). Key findings include phenological shifts in response to altered seasonality (Parmesan and Yohe 2003), adaptive evolution of thermal tolerance (Chen et al. 2018; Bennett et al. 2021), poleward range shifts (Parmesan et al. 1999; Dahms and Killen 2023), and stage-specific vulnerability to increasing temperatures (Dahlke et al. 2020). Regarding the latter, evidence is growing that the survival of early life-stages of ectotherms, such as eggs and embryos, are particularly vulnerable to changes in temperature due to narrower thermal tolerance ranges and reduced ability to escape unfavourable conditions (Kingsolver et al. 2011; Pandori and Sorte 2019; Dahlke et al. 2020). Yet, whether and how the optimal phenotype of early life-stages change with temperature remain largely unknown despite its necessity for understanding how ectothermic organisms cope with thermal variation and climate change (Liefting et al. 2010; Bautista and Crespel 2021).

The narrow thermal tolerance ranges of ectothermic early life-stages have been attributed to a higher susceptibility to oxygen limitation due to a lack of specialized tissues and structures (i.e., gills, cardio-circulatory system) (Frederich and Pörtner 2000; Pörtner and Farrell 2008; Verberk et al. 2016). Furthermore, the scope of oxygen limitation is particularly evident in aquatic environments where oxygen availability decreases with increasing water temperature at the same time as the ectothermic metabolism, and subsequently oxygen demand, increases (Martin et al. 2017).

Egg size, a key aspect of maternal investment (Mousseau and Fox 1998) and subject to a trade-off with fecundity (Roff 1992; Stearns 1992), can be important for modulating the vulnerability of aquatic ectotherms to oxygen limitation at high temperatures (Dahlke et al. 2020). This is because the area-volume ratio, and hence the capacity for oxygen diffusion, decreases with increasing egg size (van den Berghe and Gross 1989; Martin et al. 2020). The advantages of large eggs for survival, stress resilience and growth (Smith and Fretwell 1974; Anderson 1988; Heath et al. 1999; Segers and Taborsky 2011) are therefore predicted to diminish in warmer and less oxygenated waters (Bownds et al. 2010; Thorn and Morbey 2018; Martin et al. 2020), conditions expected to become more prevalent under the climate change scenario (IPCC 2022). However, empirical studies have yielded contrasting predictions, with some evidence suggesting that larger eggs may perform equally well or even better at higher temperatures or under reduced oxygen availability (Einum et al. 2002; Hall et al. 2023). As a result, firm evidence for a general decrease in the adaptive value of egg size with increasing temperature is still lacking. This limits our understanding of how thermal variation and climate change may impact egg survival and ultimately population dynamics and life-history evolution (Carter and Sheldon 2020; Rebolledo et al. 2020).

Here, we first assessed whether and how egg size varies with reproductive timing (*proxy of* temperature-at-reproduction) in a wild population of anadromous *Esox lucius* pike, an established model organism in ecology and evolution (Forsman et al. 2015). Next, we experimentally tested whether and how the adaptive value of egg size in producing viable larvae change with increasing temperature according to the theory of oxygen limitation. To do this, we conducted a split-brood experiment where artificially fertilized eggs from 75 unique families were incubated across a temperature gradient spanning the typical natural range of temperature-at-reproduction (6–14 °C), as well as an elevated temperature (18 °C) that is currently rare during spawning (Fig. S1) but is likely to become more frequent under future climate warming. We predicted that egg size would decrease with increasing temperature in nature and that this pattern would be reflected in the experiment with larger eggs outperforming smaller eggs at low temperatures but that this survival advantage of large eggs would diminish with increasing temperatures.

## Materials and methods

### Study system

This study was conducted with pike from a genetically isolated anadromous population (Nordahl et al. 2019) that migrates from the Baltic Sea to reproduce in a small creek and adjacent wetland near Mönsterås, southern Sweden (57.071615, 16.527546). Pike is well-suited for studies on egg/embryo performance across environments due to established protocols for artificial fertilization, enabling experimental crossings (families) to be split across environmental gradients (Tibblin et al. 2015; Sunde et al. 2018, 2019), but the impact of egg size on thermal performance have previously never been investigated. Moreover, pike is a eurythermal, total-spawning (the female lay all her eggs within a few days), iteroparous predator where both egg size and temperature at reproduction vary considerably among individuals within populations (Craig 1996; Berggren et al. 2016; Tibblin et al. 2016). This variation occurs despite that pike originating from the same population can share a thermal regime prior to breeding (Flink et al. 2023).

### Field sampling

During the reproductive seasons of 2021–2024, we collected 348 individual egg samples representing 262 ovulating females (some were recaptured across years) to assess how egg size varied with spawning temperature in nature (Table 1). The focal fish were captured while spawning using a stream-wide fyke net that was in place and checked every morning for ovulating females during at least 30 days per season within the given time periods (Table 1). Following capture, the females were dry stripped of approximately 20 ml of eggs that were placed on ice, to standardize conditions prior to determining egg size and performing artificial fertilizations, until reaching the laboratory facility (see below). Each female was also photographed on the left side while positioned flat alongside a reference scale to enable standardized measurements of standard lengths (SL). These measurements were obtained by digitizing landmarks using the software TpsDig (version 2.31) and extracting the data with R. Prior to being released back to the river, females were tagged with Passive Integrated Transponders (HDX23, Biomark, Boise, Idaho, USA) and (for most fish) with an acoustic transmitter (MP13, THELMA BIOTEL, Norway) to enable individual identification upon recapture and future studies of movement behavior respectively. Two temperature data loggers (HOBO Pendant Temperature/Light 64 K, Onset Computer Corporation) were deployed at the sampling site throughout each spawning season to monitor the natural temperature range during spawning.

**Table 1:** A total of 348 egg samples were stripped from 262 ovulating females during four consecutive spawning seasons from 2021 to 2024, with some females being sampled over multiple years. The temperature range is based on the mean daily ambient water temperature at the sampling site during the capture periods, as recorded by temperature loggers

Year	Capture period	Temperature range	Body size (SL cm, mean ± SD)	*N*_females_
2021	April 3 – May 20	5.6–15.6 °C	67.5 ± 11.2	91
2022	March 28 – May 12	3.9–13.9 °C	66.7 ± 10.0	142
2023	April 11 – May 14	7.6–15.5 °C	70.0 ± 8.8	85
2024	April 10 – May 01	9.1–21.2 °C	69.7 ± 8.6	30

For the split-brood experiment (described below), we specifically sampled eggs from a total of 75 ovulating pike females (size range: 41.8–94.8 cm SL, mean ± SD: 66.9 ± 10.8 cm) during the reproductive season of 2022. These individuals represented a, across size and time, stratified subsample from a total of 142 ovulating females sampled during the 2022 season (Table 1). To conduct artificial fertilizations for the split-brood experiment, we also sampled milt from a corresponding number of randomly selected males that were also put on ice during transport to the laboratory.

### Quantification of egg size

To determine egg size, a subsample of 1 ml of eggs from the initial (20 ml) egg sample were taken for each female (see above), spread as a single layer in a petri dish, and photographed to determine the number of eggs using Image J (Schneider et al. 2012). The eggs were then dried at 60 °C overnight before being weighed on a precision scale (Kern^®^ precision Analytical balance, model ABJ 220-4NM). Egg size for each female was subsequently determined by dividing the total dry weight of 1 ml egg sample with the total number of eggs (range across females in the split-brood experiment: 1.5–3.2 mg/egg; mean ± SD: 2.4 mg ± 0.3; range across all females: 1.4–3.6 mg/egg, mean ± SD: 2.5 mg ± 0.3). Dry weight were used as proxy of egg size (volume) as it is well-established that these measurements are correlated (Stuart et al. 2020), which was also confirmed in our system based on volume and dry weight measurements for 29 females (Pearson correlation: *r* = 0.52, *t* = 3.13, *p* = 0.004).

### Split-brood experiment to test how temperature influences egg size-dependent offspring survival

We evaluated whether and how egg size influences temperature-dependent survival by conducting a split-brood experiment where eggs (full-sibs) of the 75 families of pike were incubated at four temperatures (mean ºC ± SD based on duplicated HOBO temperature loggers in each treatment: 6.4 ± 0.6; 10.1 ± 0.5; 13.6 ± 0.7; 17.6 ± 0.4; hereafter referred to as the 6, 10, 14 and 18 °C treatments) using a full-factorial nested design with two replicates per family and temperature treatment, resulting in a total of 600 experimental units (Fig. S2). Treatments were chosen to include temperatures (6, 10, 14 °C) corresponding to the previously documented main in situ range of temperatures at reproduction in the focal area (which was also confirmed by this study, Table 1) (Sunde et al. 2019), as well as the upper extreme of the observed spawning temperature (18 °C, Fig. S1) that currently occurs rarely but is projected to become more frequent in the study region under climate warming (Zalewska et al. 2024). Temperature treatments were achieved through incubating the eggs in four temperature-controlled mesocosm rooms in the laboratory facility.

The focal experimental families were produced through artificial fertilization of in situ dry-stripped gametes of randomly assigned female-male pairs (each female and male only used for one unique family) following the established method for the focal species (Sunde et al. 2018, 2019). In short, each experimental replicate was produced independently by placing eggs (ca. 35/replicate, taken from the 20 ml egg sample from each focal female) and milt (in excess, about 200 µl/replicate) in a small porcelain bowl, followed by the addition of temperature treatment-specific water (approx. 1 ml) and a gentle swirl to allow mixing. Eggs were then let to rest for 120 s before excess milt was removed by rinsing the eggs three times with treatment-specific water. Immediately after rinsing, the eggs were gently transferred to a batch system were each independent unit consisted of two 0.8 L containers, one intact with another one inside where the bottom had been replaced by a plastic fine mesh (1.5 mm), filled with treatment-specific water, and randomly positioned in custom-made racks. This system allows non-invasive water changes without disturbing eggs and embryos. Treatment-specific water was produced by first allowing maturation of tap water through aeration in 18 °C for a minimum of 48 h before water was transferred to each mesocosm room and allowed to reach the designated temperature prior to distribution to the experimental set-up. The light-dark cycle and light intensity were kept constant at 14 L:10D and 30 µmol m^−2^s^− 1^ respectively throughout the experiment.

Each replicate was monitored daily throughout the experiment by manually counting the number of viable eggs/embryos. Dead pike eggs/embryos, readily identified by becoming opaque (eggs) and/or pale (embryos) (Sunde et al. 2018), were recorded before being removed. Each replicate was photographed (Canon EOS 250D DSLR) the first day after fertilization (to determine the initial number of eggs) and thereafter at each event of water exchange until the stage when embryos had consumed the egg-yolk and became free-swimming (exogenous life-stage) at which time replicates were terminated and final number of viable embryos determined. Time from start of incubation to free-swimming stage varied among the temperature treatments: 32.1 ± 3.02 (mean ± SD) days at 6 °C, 17.1 ± 2.07 days at 10 °C, 11.4 ± 1.87 days at 14 °C, and 8.29 ± 0.97 days at 18 °C. Water exchanges (50% of the volume) were conducted manually in each replicate at a fixed interval of approximately 40 degree-days meaning that its frequency increased with temperature. This was by design to mimic how oxygen availability changes with temperature but safeguarding against oxygen depletion which was confirmed by a significant negative relationship between temperature and oxygen concentrations (Linear regression, effect of temperature, *b ± SE* = -0.27 *±* 0.01, *t* = -25.6, *P* < 0.001, R^2^ = 0.75; Mean mg O^2^ L⁻¹ ± std: 6 °C = 11.6 ± 0.5; 10 °C = 10.5 ± 0.8; 14 °C = 9.2 ± 0.9; 18 °C = 8.9 ± 0.5) based on daily measurements of dissolved oxygen (WTW MultiLine Multi 3620 IDS, FDO 925 optical DO sensor) in three random replicates of each treatment. These oxygen concentrations were also well above levels previously reported to impair embryonic development in pike (e.g., ~ 50% saturation; Siefert et al. 1973).

### Statistics

To investigate whether egg size varied over the spawning season as the temperature increases, we analyzed four years of data on egg size and reproductive timing (Julian date for arrival to spawning area). Egg size was modelled with a Linear Mixed Model (LMM) using Gaussian error distribution. The initial full model included year, reproductive timing, and their interaction term as fixed effects. The model was refitted without the interaction term if this was found to be non-significant. Following the detection of a significant effect of reproductive timing on egg size, we compared the model to a refitted version including female body size, using the mediation package in R (version 4.5.1; Tingley et al. 2014) to evaluate whether the effect of reproductive timing was mediated by a seasonal decline in body size (average causal mediation effect, ACME) or whether it exerted an additional size-independent influence on egg size (average direct effect, ADE). To test whether and how egg size changed with female standard length, we modelled egg size with a LMM and included standard length as fixed effect. All models included a random intercept for individual ID to account for repeated measures of the same individuals across years. Model fitting, assumption testing, and evaluation of fixed effects were conducted using the same packages and techniques as described for the GLMM (see below).

We used Generalized Linear Mixed Model (GLMM) to evaluate effects of egg size on temperature-dependent survival. Egg survival, defined as the ratio of offspring alive after yolk-sac absorption, was modeled using a binomial distribution and a logit transformation function. Egg size and treatment temperature were included as continuous fixed effects, along with their interaction, to assess whether the effect of egg size on survival varies along the temperature gradient utilized in the experiment. Additionally, an interaction between the spawning time and treatment temperature was included as a fixed effect to control for potential timing-induced impacts on performance curves (Hall et al. 2021). Random intercepts and slopes for temperature treatment were included for each female to account for the non-independence of repeated measures within females and to allow for individual-level variation in temperature response.

Julian date was used as the predictor variable both when modeling egg size variation in the field and egg survival in the experiments, serving as a proxy for the overall seasonal increase in temperature. This choice was made to capture the overall seasonal progression of rising temperatures, rather than relying on point estimates or mean values of temperature over arbitrary time windows that are influenced by short‑term stochastic fluctuations within days and years (Fig. S1). Such fluctuations do not necessarily correspond to the temperature cues that influence females’ reproductive timing or to the thermal conditions experienced by offspring, owing to potential temporal and spatial mismatches between the temperature measurements and the actual temperatures encountered by the relevant life stages of the fish. However, the increased likelihood of encountering higher temperatures at later Julian dates is general across years and consistent within different parts of the spawning habitat.

The significance levels of the fixed effects were estimated using type III Wald Chi-square test. For random effects, the significance levels were determined using Likelihood Ratio Tests (LRT). We specifically evaluated the random intercept for female identity, the random slope with temperature treatment for female identity, and their correlation. This evaluation was conducted through stepwise comparisons of models with and without the random term of interest. Families with less than 5% survival rate across temperature treatments were excluded from all the analyses due to the non-informative nature of the data to accurately estimate performance curves and potentially bias the results. Similarly, one female with an outlier value for egg size - length relationship was excluded from the analysis, leaving 64 families from the initial 75 for the analyses. Presence of outliers was tested using studentized residuals and Bonferroni-corrected *p*-values. All models (GLMM and LMM) were fitted using the glmmTMB package (v. 1.1.10; Brooks et al. 2017), except for the models being compared by the mediation package, that were fitted using lme4 (v. 1.1–35.5; Bates et al. 2015). Model assumptions, including assessment of dispersion and outlier detection, were tested and assessed using functions from the package DHARMa (v. 0.4.6; Hartig 2022). A random intercept for each replicate (i.e., observation-level random effects) nested within individuals was included in the full model to mitigate problems with overdispersion (Harrison 2015). To evaluate potential issues with multicollinearity, we tested for correlations between reproductive timing and egg size but found no indications of such issues (*r* < 0.1).

All statistical analysis and visualization of results were performed using R version 4.4.1 (RCoreTeam 2024).

## Results

### Females in the wild lay smaller eggs in warmer water

**Fig. 1:**
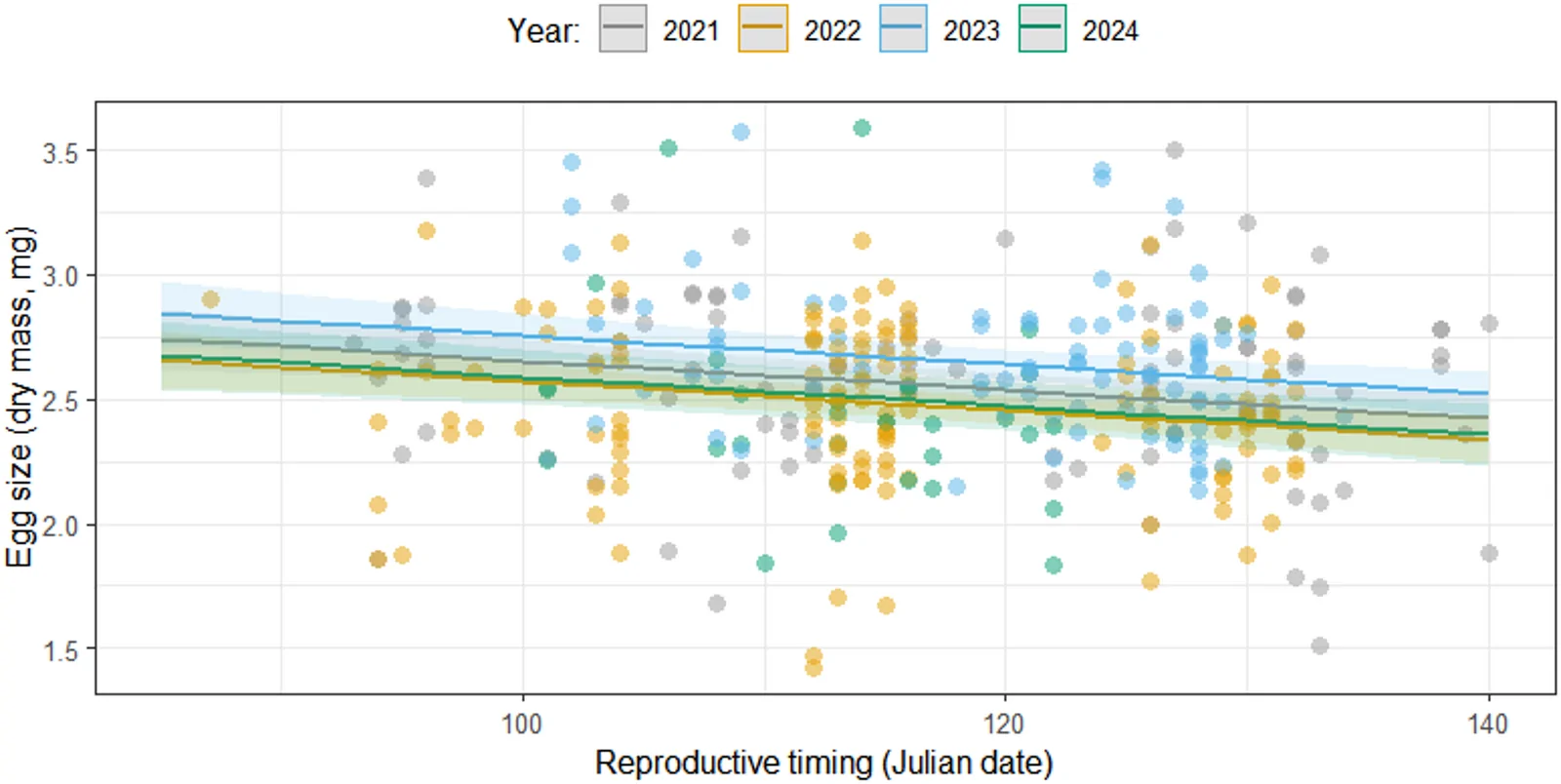
Egg size decreased over the spawning seasons, with females (*N* = 348) arriving later producing smaller eggs. While the slope did not differ between years, there was a significant main effect of year, indicating that egg size varied among years independently of reproductive timing

Females that spawned later in spring, at higher temperatures, produced smaller eggs (LMM, effect of reproductive timing, N_individuals_= 262, N_observations_= 348, estimate ± SE = -0.006 ± 0.0017, χ² = 12.1, *P* < 0.001, Fig. 1). The decrease in egg size over the season was accompanied by a decrease in female size (LMM, effect of length on reproductive timing, estimate ± SE = -0.2 ± 0.06, *df* = 259, *t*=-3.2, *P* = 0.002), with early arrival of larger females with larger eggs, while later spawners were smaller females with smaller eggs. Model comparison confirmed that the effect of reproductive timing on egg size was explained by seasonal changes in female size (ACME [95% CI] = -0.01 [-0.018; -0.004], *P* < 0.001), while timing itself had no size-independent effect (ADE [95% CI] = -0.025 [-0.059; 0.008], *P =* 0.16). Egg size also varied among years (main effect of year χ² = 30.9, *P* < 0.001, Fig. 1), albeit independently of reproductive timing (effect of interaction between reproductive timing and year, χ² = 2.8, *P* = 0.42), suggesting that the decrease in egg size over the spawning season was consistent among years.

### Large eggs lose the survival advantage as temperature increases

**Fig. 2:**
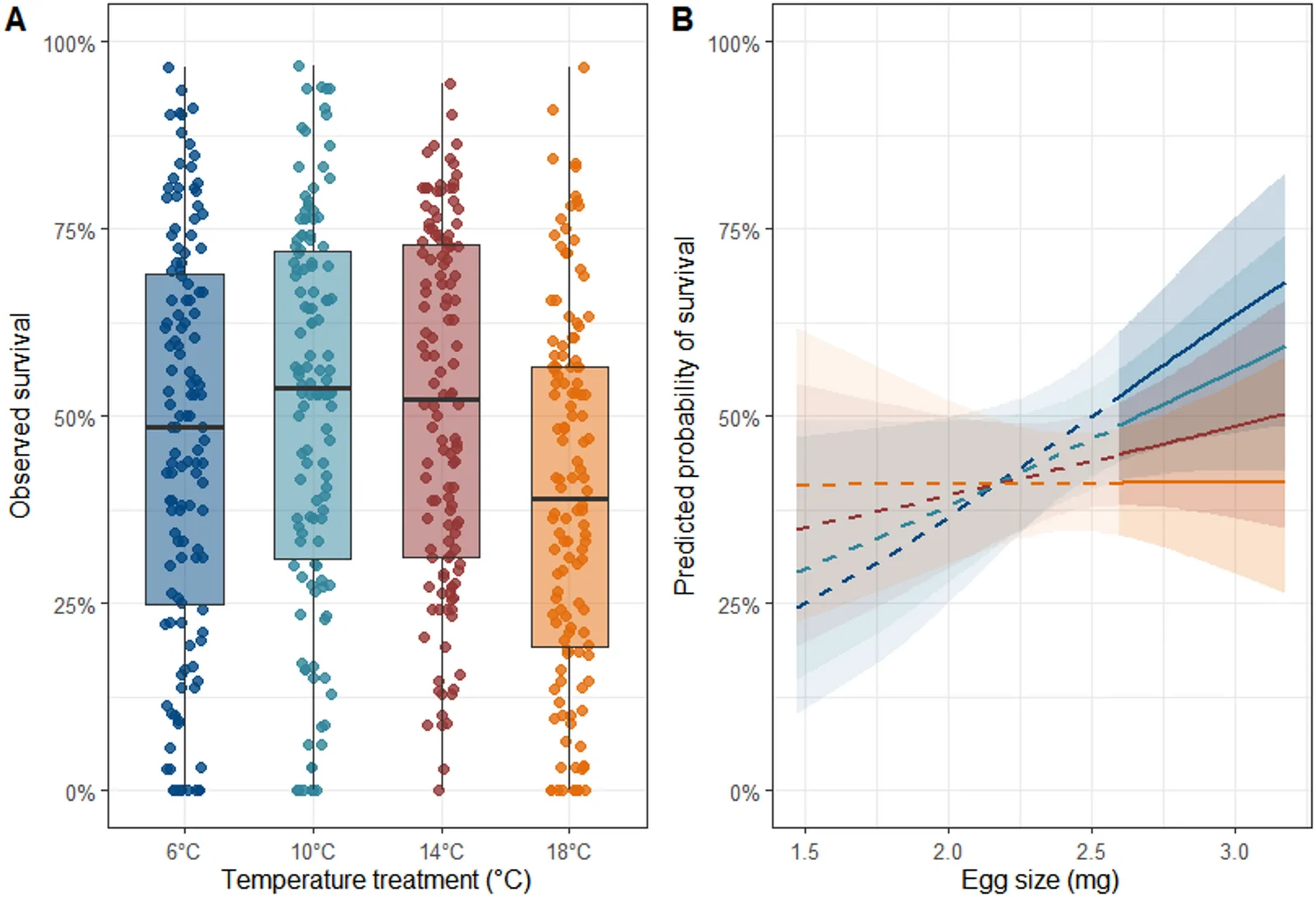
**(A)** Observed offspring survival (%) per replicate (~ two replicates per temperature treatment and female, *N* = 64) across the four temperature treatments. Each point represents a single replicate and is jittered along the x‑axis to reduce overlap, while boxplots summarize the distribution within each treatment by showing the median, first and third quartiles, and whiskers extending to the minimum and maximum observed values. **(B)** Predicted probabilities of offspring survival as a function of egg size across the four temperature treatments. This significant interaction illustrates the temperature-dependent effect of egg size on the probability of survival. Larger egg sizes had increased survival at lower temperatures, but this positive relationship diminished with increasing temperature, approaching zero at higher temperatures. The post-hoc estimated marginal means comparison test performed on the model, revealed not statistically significant differences among temperature treatments where the egg size was < 2.6 mg. Solid lines indicate regions where pairwise temperature contrast are statistically significant ( egg size ≥ 2.6 mg; *P* < 0.05) while dashed lines indicated the region where egg size was under the non-significance region. Shaded regions indicate the confidence intervals for the predictions

Survival rates varied among treatments across families with 46.7% ± 27% survival at 6 °C, 50.8% ± 26% at 10 °C, 51.3% ± 24% at 14 °C, and 39.3% ± 25% at 18 °C (mean ± SD, Fig. 2A). The effect of temperature on survival was dependent on egg size (GLMM, egg size x temperature treatment, *χ*^*2*^ = 5.70, *P* = 0.017, Table 2; Fig. 2B), implying that the slope of the thermal performance curve varied with egg size. Specifically, survival increased with egg size in colder, well-oxygenated water (Estimated marginal means of linear trends, 6 °C, slope ± SE = 1.10 ± 0.51) but this size-effect diminished as temperature increased (10 °C, slope ± SE = 0.74 ± 0.43; 14 °C, slope ± SE = 0.37 ± 0.40). At higher temperatures, egg survival appeared to be independent of size (18 °C, slope ± SE = 0.01 ± 0.43), indicating a critical temperature threshold beyond which the size-dependent survival advantage is lost.

**Table 2:** Model output from the full Generalized Linear Mixed Model (GLMM), presented separately for fixed and random effects. The table includes results from the Type III Wald chi-square (χ²) test of deviance. The response variable, survival, was modeled with a binomial outcome (survival/replicate)

Fixed effects	Estimate	SE	χ²	Df	*P*
(Intercept)	0.91	2.44	0.11	1	0.708
Temperature	-0.20	0.14	2.00	1	0.151
Egg size	1.65	0.67	5.97	1	**0.014**
Spawning day	-0.04	0.017	6.09	1	**0.011**
Temperature: Egg size	-0.09	0.038	5.69	1	**0.017**
Temperature: Spawning day	0.003	0.001	13.05	1	**< 0.001**
**Random Effects**	**Variance**	**SD**			**Correlation**
Female (intercept)	2.03	1.43			
Female (slope for temperature)	0.004	0.062			-0.75
Replicate (intercept)	0.66	0.81			
**Observations**	**N**				
N_Females_	64				
N_Observations_	510				
***R*** **²**	**Value**				
Marginal	0.05				
Conditional	0.605				

Including the full random structure with a random intercept for individual identity and a random slope for temperature significantly improved the model fit over the model with only a random intercept (LRT; χ² = 10.7, *P* = 0.005), yielding the lowest AIC compared to other models with simplified random structure (ΔAIC > 6). This indicates that after conditioning for egg size, there is individual variation in the response by females to the temperature treatment (i.e., a significant female-by-temperature interaction effect). Moreover, the results showed that egg size was positively associated with female length (LMM, effect of standard length, *b* ± *SE* = 0.018 ± 0.002, *χ*^*2*^ = 102.11, *P* < 0.001; Fig. S3).

## Discussion

Environments are warming due to ongoing climate change, a trend expected to accelerate over the coming decades with profound consequences for population viability and species persistence (IPCC 2022). Early life-stages of aquatic ectotherms, such as fish eggs and embryos, are particularly sensitive to rising temperatures, yet the mechanisms underlying variation in their thermal performance remain poorly understood (Dahlke et al. 2020).

In this study, we combined four years of field observations to assess how egg size varies within reproductive season (i.e., across the seasonal thermal gradient) with a split-brood thermal gradient temperature experiment conducted to evaluate whether and how the adaptive role of egg size changes with temperature. Field data showed that egg size declined over the spawning season as a consequence of decreasing female size, while the experiment revealed an interactive effect of egg size and temperature on survival, with the expected advantage of larger eggs diminishing as temperatures increased, likely due to size-dependent oxygen limitation. Together, our findings enhance our mechanistic understanding of the adaptive value of egg size under changing thermal environments and suggest that warming may indirectly shape population demographics and reproductive allocation strategies.

Egg size and incubation temperature are critical factors modulating the reproductive success of ectotherms (Brooks et al. 1997; Huey and Berrigan 2001), yet their interactive effects remain underexplored. This is surprising given substantial evidence from both inter- and intra-specific studies demonstrating that females breeding at lower temperatures tend to produce larger eggs, whereas higher temperatures are often associated with smaller eggs (Yampolsky and Scheiner 1996; Bownds et al. 2010; Feiner et al. 2016; Barneche et al. 2018). A similar pattern emerged in our study, with egg size declining over the spawning season as temperature increased. However, this pattern was likely caused by large females with larger eggs spawning earlier. From an adaptive perspective, this may reflect adaptation to the thermal conditions experienced during embryonic development (Feiner et al. 2016; Hall et al. 2021). Alternatively, larger and more competitive females may spawn earlier due to general fitness advantages of early reproduction, such as priority effects, as documented in many species or a combination of the two (Einum and Fleming 2000; Ward et al. 2006). The former interpretation aligns with predictions from the oxygen limitation hypothesis (Martin et al. 2020), yet this hypothesis has rarely been tested explicitly across ecologically relevant temperature gradients (Bownds et al. 2010; Anderson and Gillooly 2020).

We tested whether and how the adaptive role of egg size changes with temperature through a temperature gradient split-brood experiment. This uncovered that the effect of egg size on early life-stage survival varies with temperature. Specifically, we found that larger eggs were more successful in producing viable larvae at low temperatures, consistent with the well-established ‘bigger-is-better’ hypothesis (Smith and Fretwell 1974; Anderson 1988; Brooks et al. 1997; Stuart et al. 2020). However, this advantage diminished with increasing temperature within the current natural spawning temperature range (6–14 °C) and was entirely absent at an increased temperatures (18 °C) that may arise in the future as the climate continues to change (Zalewska et al. 2024). Our experimental results are consistent with the hypothesis that, as larger eggs develop and their oxygen demand increases, their lower oxygen diffusion efficiency, arising from their lower surface area to volume ratio compared to smaller eggs, causes them to suffer more from oxygen limitation in warmer, and less oxygenated water than smaller eggs (Martin et al. 2017). While the potential for larger fish eggs to be less successful at higher temperatures due to oxygen limitation has been proposed (Thorn and Morbey 2018; Martin et al. 2020), as has the opposite pattern of larger eggs performing better in warmer and/or less oxygenated water (Einum et al. 2002; Hall et al. 2023), previous studies have relied on either modeling approaches or not evaluated the combined effect of temperature and oxygen availability. In this context, our study provides rare empirical support to the notion that larger eggs may lose their inherent fitness advantage in a warming world and that this may be due to oxygen limitation.

The ecological and evolutionary consequences of egg size being linked to temperature-dependent survival hinge on the underlying causes of egg size variation. Previous research has demonstrated that egg size variation can involve both genetic components (Heath et al. 2003), and maternal effects through environmentally induced phenotypic plasticity (Heath et al. 1999) and developmental plasticity (Berggren et al. 2016). For instance, it is well established that egg size often is positively correlated with female age and size in both fish (Berggren et al. 2016; Rollinson and Rowe 2016; Hall et al. 2023) and other vertebrate taxa (Lim et al. 2014). This is also supported by our data, which revealed a positive relationship between female size – also a reliable proxy of age for the focal species (Tibblin et al. 2015) – and egg size indicative of developmental plasticity. In contrast, environmentally induced phenotypic plasticity seems less likely to contribute significantly to egg size variation in our system, as pike typically aggregate in a confined sympatric habitat for several months before spawning (Flink et al. 2023). Regarding genetic components of egg size variation, this could not be formally evaluated in the current study. However, the significant female-by-temperature interaction effect suggests that there is genetic variation for developmental plasticity or thermal plasticity of eggs to evolve as a response to a warming climate (Forsman 2015).

Given that the inherent advantage of large eggs may, based on our results, be diminished in the future scenario of warmer and less oxygenated aquatic environments, adaptive evolution could favor a shift in allocation strategies toward females producing smaller but higher number of eggs (Roff 1992; Stearns 1992). This would, of course, require that egg size variation includes genetic components as well as that egg size dependent offspring survival is impacting individual fitness (Roff 1992; Stearns 1992; Bernardo 2015). Similarly, this scenario could relax selection for large females, given their association with producing large eggs, which could ultimately reinforce the well-documented pattern that increasing temperatures favor smaller body sizes (e.g., the temperature-size rule) (Angilletta et al. 2004; Daufresne et al. 2009; Sheridan and Bickford 2011; Cheung et al. 2013; Pettersen et al. 2019; Salvatteci et al. 2022). However, the extent to which such evolutionary shifts in allocation strategies and body size are realized will also depend on whether phenological responses can buffer early life stages from warming conditions. Specifically, buffering would only occur if the timing of reproduction can adjust to shifts in seasonality driven by climate change, thereby maintaining an alignment between larger eggs produced by large females and the lower temperatures experienced early in the season. For such alignment to persist, individuals and populations would need to track advancing thermal regimes through corresponding shifts in reproductive timing. While temperature is an important cue shaping reproductive timing in fish, reliance on additional cues such as photoperiod may constrain the capacity of populations to rapidly adjust their timing of reproduction to shifting seasonal thermal conditions (Woods et al. 2022). In that case, the decline in survival of large eggs in warmer water can indirectly impact population dynamics. This is because large eggs typically produce larger and more successful larvae with higher stress resistance and survival probability (Anderson 1988; Salin et al. 2015). Loss of these high quality larvae from future recruitment cohorts could weaken population resilience towards predation (Nilsson et al. 2019) and other anthropogenic stressors such eutrophication (Dietz et al. 2021).

To conclude, this study provides empirical support for the hypothesis that the advantage of large fish eggs may diminish in a warming world possibly because of oxygen limitation. This could lead to evolutionary modifications in life-history strategies, including changes in the egg size – fecundity trade-off, and contribute to shifts in population dynamics. In this context, preserving genetic and phenotypic variation both among and within populations is crucial for maintaining adaptive potential and safeguarding populations against the impacts of climate change. Future studies should aim to evaluate the generality of our findings by examining the link between egg size, oxygen concentration, and temperature in early life-stage survival across a broad range of species, as well as identifying potential genetic components of intraspecific egg size variation and evaluating how egg size variation may aid population resilience towards altered thermal environments.

## Supplementary Information

Below is the link to the electronic supplementary material.

Supplementary Material 1

## Data Availability

The data and code that support this study are uploaded and available on the DRYAD repository (10.5061/dryad.brv15dvkh).
