# Supplementary material for: Large fish eggs may lose their edge as temperature rises
Source: Oecologia. 2026 Jul 10;208(8):91. doi: 10.1007/s00442-026-05932-3 (PMC13354630; doi:10.1007/s00442-026-05932-3)
Supplement: Supplementary file 1 — Supplementary Material 1 [file 442_2026_5932_MOESM1_ESM.docx]

**ELECTRONIC SUPPLEMENTAL MATERIAL FOR:**

**Large fish eggs may lose their edge as temperature rises**

Author list: Francesca Leggieri^1^, Oscar Nordahl^1^, Markus Zöttl^1^, Hanna Berggren^1^, Petter Tibblin^1^*

Affiliations: 1Centre for Ecology and Evolution in Microbial Model Systems (EEMiS), Department of Biology and Environmental Science, Faculty of Health and Life Sciences, Linnaeus University, SE-39231 Kalmar, Sweden

*Corresponding author: Petter Tibblin, petter.tibblin@lnu.se, Linnaeus University, Stuvaregatan 4, 391 82 Kalmar, Sweden. Phone: +46 480 446745

**Figure S1**.

Hourly mean water temperature (°C) recorded at the capture site during the spawning period (Julian day) for the years 2021-2024. Each coloured line represents a different year and the dotted line indicates mean daily temperature increase across years


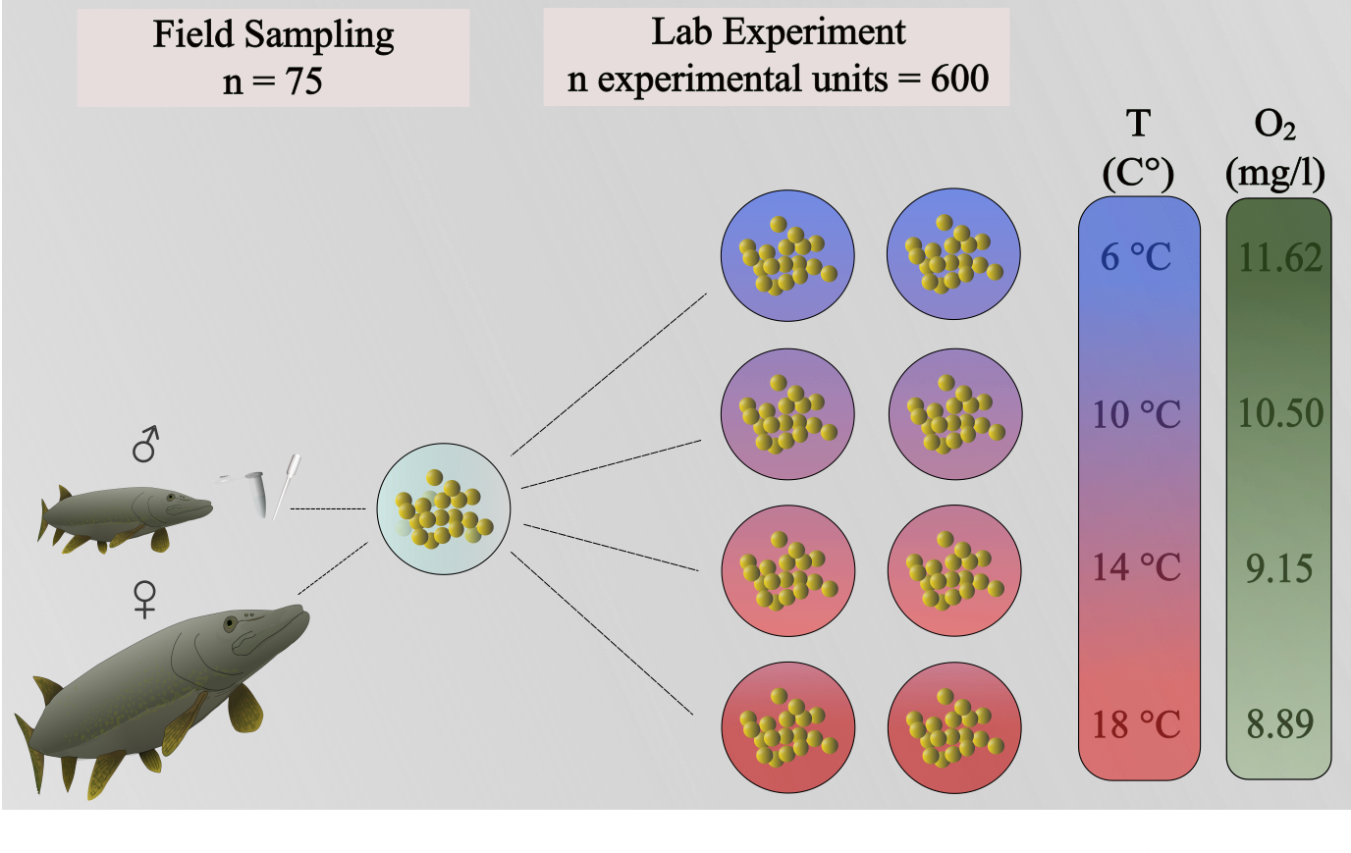


**Figure S2**. The experimental split-brood study design including temperature treatments and associated oxygen concentrations (mean O_2_/treatment across the experimental duration). Eggs and milt were sampled from females and males, respectively, in the spawning area. The temperature treatments were conducted in the laboratory


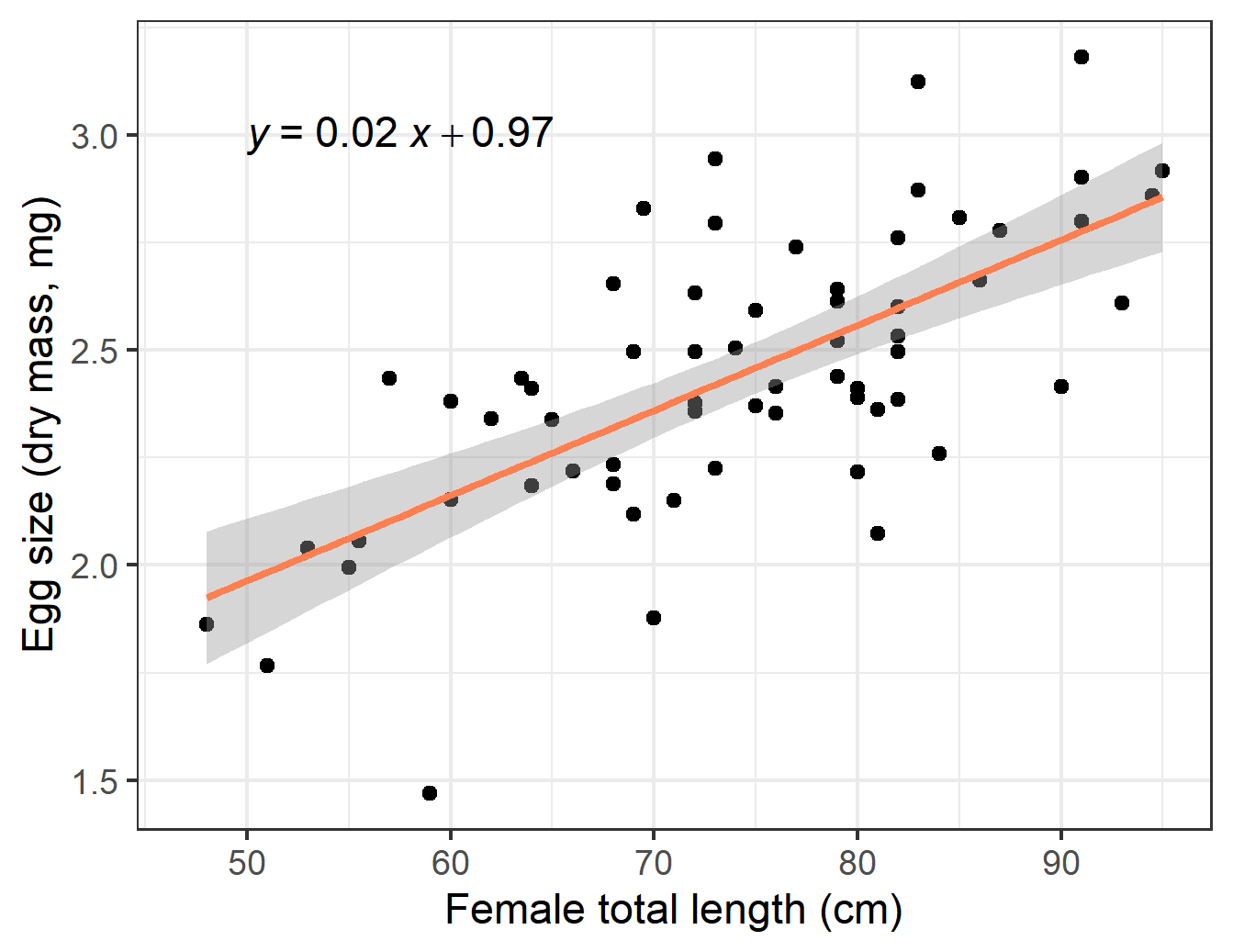


**Fig. S3.** Observed linear relation across female body size and egg size (R2 = 0.46, N=64). This relation indicates that egg size increases with body size with a predicted increase of 0.019821mg per each cm of body size
